# Health Costs of Older Opioid Users with Pain and Comorbid Hypercholesterolemia or Hypertension in the United States

**DOI:** 10.3390/diseases9020041

**Published:** 2021-06-10

**Authors:** David R. Axon, Srujitha Marupuru, Shannon Vaffis

**Affiliations:** College of Pharmacy, The University of Arizona, Tucson, AZ 85721, USA; marupuru@pharmacy.arizona.edu (S.M.); vaffis@pharmacy.arizona.edu (S.V.)

**Keywords:** opioid, pain, hypercholesterolemia, hypertension, health expenditures, cross-sectional studies

## Abstract

This retrospective cross-sectional database study used 2018 Medical Expenditure Panel Survey data to quantify and assess differences in healthcare expenditures between opioid users and non-users among a non-institutionalized sample of older (≥50 years) United States adults with pain in the past four weeks and a diagnosis of comorbid hypercholesterolemia (pain–hypercholesterolemia group) or hypertension (pain–hypertension group). Hierarchical multivariable linear regression models were constructed by using logarithmically transformed positive cost data and adjusting for relevant factors to assess cost differences between groups. Percent difference between opioid users and non-users was calculated by using semi-logarithmic equations. Healthcare costs included inpatient, outpatient, office-based, emergency room, prescription medication, other, and total costs. In adjusted analyses, compared to non-users, opioid users in the pain–hypercholesterolemia and pain–hypertension groups respectively had 66% and 60% greater inpatient expenditure, 46% and 55% greater outpatient expenditure, 67% and 72% greater office-based expenditure, 50% and 60% greater prescription medication expenditure, 24% and 22% greater other healthcare expenditure, and 85% and 93% greater total healthcare expenditure. In conclusion, adjusted total healthcare expenditures were 85–93% greater among opioid users versus non-users in older United States adults with pain and comorbid hypercholesterolemia or hypertension. Future research is needed to identify opioid use predictors among these populations and reduce expenditures.

## 1. Introduction

Older adults in the United States (US) commonly have to manage multiple chronic diseases [1,2]. In particular, the prevalence of hypercholesterolemia (12.5%) and hypertension (63.1%) is high for US adults aged 60 and older [3,4]. Furthermore, it has been estimated that 60–75% of older adults (aged 65 years and older) in the US experience some level of persistent pain [5]. Prevalence estimates for US adults aged 50 and older, when many people start to experience chronic conditions [6], are not readily available. However, these figures demonstrate an encumbrance on the adult population that often results in disability, frequent physician visits, medication use, poorer health outcomes, and poorer quality of life [7,8].

Opioid medications are commonly used for the management of pain, and multiple studies have reported that opioid use in the US has increased in recent years. A systematic review by Axon et al. demonstrated that older adults employ numerous strategies for chronic pain management, including opioids, non-steroidal anti-inflammatory drugs, and non-pharmacological methods [9]. Likewise, data from a national survey on hospital and other ambulatory care in the US showed that opioid use more than doubled from 4.1% to 9.0% among adults aged 65 and older in recent years [10].

Several studies have shown increased costs associated with management of chronic conditions, including pain, among older US adults. One recent study of older US adults with pain demonstrated that the adjusted total healthcare costs of those who were prescribed opioids were 105% greater than those who were not prescribed opioids [11]. Another study showed costs have increased by as much as 41% in the treatment of hypercholesterolemia in US adults [12], while a further study estimated additional costs of $2000 per person associated with treatment of hypertension in US adults [13]. Pain also has significant economic consequences, for both patients and society, with total costs ranging from $560 billion to $635 billion per year in 2010 [7,14].

Although previous work has explored the interaction of chronic disease and costs [15], there is a lack of research investigating expenditures associated with opioid use among older US adults with pain and specific common comorbid conditions such as hypercholesterolemia and hypertension. These conditions were chosen due to their relatively common prevalence in the US (and their subsequent impact on healthcare utilization and expenditures). It is critical to understand the costs associated with managing these conditions in conjunction with other chronic conditions, such as pain, given that they are common and treatable risk factors for leading causes of death (such as heart attack and stroke) [16,17].

Therefore, the objective of this study was to quantify and assess differences in healthcare expenditures between opioid users and non-users among a nationally representative sample of older US adults (≥50 years) with pain and comorbid hypercholesterolemia or hypertension.

## 2. Materials and Methods

*Study design and data:* This study used the most currently available data (2018) from the Medical Expenditure Panel Survey (MEPS) and followed a retrospective cross-sectional study design. MEPS uses the sampling framework of the previous years’ National Health Interview Survey (NHIS) and generates nationally representative estimates by oversampling disabled and minority groups in the US. MEPS data are obtained from several interview panels conducted over a two-year period. The MEPS household component (MEPS-HC) compiles an abundance of self-reported data for all household members, including data on personal characteristics, chronic health conditions, prescription medications, and healthcare costs [18].

*Study populations:* Data from the 2018 full-year consolidated data file were used to develop two study groups (a pain–hypercholesterolemia group and a pain hypertension group), using the following inclusion criteria: (1) alive for the full calendar year, (2) aged 50 years or older, (3) reported having pain in the past four weeks, (4) a diagnosis of hypercholesterolemia (for the pain–hypercholesterolemia group) or a diagnosis of hypertension (for the pain–hypertension group), and (5) positive total healthcare expenditures. Pain was identified according to responses to the following item: “During the past 4 weeks, pain interfered with normal work outside the home and housework”. Subjects were deemed to have pain if they responded “a little bit”, “moderately”, “quite a bit”, or “extremely”. Meanwhile, subjects were excluded if they responded “not at all” [19,20,21].

*Independent variables:* Opioid use status (opioid user or non-user) served as the key independent variable. Opioid use was identified by the presence of at least one Multum Lexicon therapeutic class code for narcotic analgesics or narcotic analgesic combinations in the 2018 prescribed medicines file [22,23].

The following variables (conceptualized according to the Andersen Behavioral Model) served as covariates in statistical models [24]. (1) Predisposing factors included age (50–64, ≥65 years), gender (male, female), and race (white, other); (2) enabling factors included education (high school or less, higher than high school), employment (employed, unemployed), health insurance (private, public, uninsured), and poverty status (split at the 200% federal poverty level; poor/near poor/low income, middle/high income); (3) need factors included number of chronic conditions (these chronic conditions were selected because of their relatively high prevalence and availability of clinical care guidelines: angina, arthritis, asthma, cancer, chronic bronchitis, coronary heart disease, diabetes, emphysema, hypercholesterolemia, hypertension, joint pain, myocardial infarction, other unspecified heart disease, and stroke; hypertension and hypercholesterolemia were not included in their respective study groups; <5 conditions, ≥5 conditions), instrumental activities of daily living (IADL) limitations (yes, no), activities of daily living (ADL) limitations (yes, no), functional limitations (yes, no), work limitations (yes, no), visual limitations (yes, no), hearing limitations (yes, no), pain severity (little, moderate, quite a bit, extreme), and perceived health status (excellent/very good/good, fair/poor); (4) the only personal health practices factor was exercise (≥30 min of at least moderate intensity physical exercise five times a week; yes, no); (5) the only external environmental factor was census region (Northeast, Midwest, South, West) [20,21].

*Healthcare expenditures:* This study assessed seven healthcare expenditure outcomes: (1) inpatient, (2) outpatient, (3) office-based, (4) emergency room, (5) prescription medication, (6) other, and (7) total expenditures. MEPS expenditure data include direct payments for healthcare provided during the year by private insurance, Medicaid, Medicare, out-of-pocket, and other sources, but not indirect payments or over-the-counter medication expenses. Inpatient expenditures included all hospital inpatient visits regardless of length of stay and instances where patients were admitted to hospital after presenting to the emergency room. Outpatient and office-based expenditures included visits to physicians and non-physician providers. Non-physician providers included chiropractor, midwife, nurse, nurse practitioner, optometrist, podiatrist, physician’s assistant, physical therapist, occupational therapist, psychologist, social worker, technician, and receptionist/clerk/secretary, among others. Inpatient, outpatient, and emergency room expenditures were inclusive of facility expenses and separately billing doctor expenses (payments made to physicians for services provided but billed separately). Prescription medication expenditures included initial and refill out-of-pocket and third-party costs. Other expenditures included dental care, vision care, home healthcare, and other medical equipment and services. Total expenditure included all healthcare service expenditures for subjects in 2018 [20,21].

*Data analysis:* All variables of interest were available to the research team via MEPS Public Use Files (PUFs). National estimates were obtained by accounting for the complex survey design of MEPS and using the appropriate weighting variables provided by MEPS. Comparisons were made between each independent variable and opioid use status, using chi-square tests for categorical variables (characteristics of subjects) and t-tests for continuous variables (descriptive healthcare expenditures) in both the pain–hypercholesterolemia and pain–hypertension group. To assess differences in healthcare expenditures between opioid users and non-users, hierarchical linear regression models were constructed by using logarithmically transformed cost data (due to the non-linear nature of healthcare cost data). An unadjusted model that assessed the association of opioid use status on the dependent variable was constructed first, followed by a series of multivariable models that adjusted for an additional group of factors (predisposing, enabling, need, personal health practices, and external environmental) each time. That is, Model 1 was unadjusted; Model 2 contained the predisposing factors; Model 3 contained the predisposing and enabling factors; Model 4 contained the predisposing, enabling, and need factors; Model 5 contained the predisposing, enabling, need, and personal health practices factors; and Model 6 contained the predisposing, enabling, need, personal health practices, and external environmental factors. The results of the fully adjusted models (i.e., Model 6) are presented in the result section. Variance estimates were calculated by using the Taylor-series linearization method. Percent difference was calculated by using semi-logarithmic equations (e^β^−1) and represents the difference in expenditure between opioid users and non-users [25]. The assumptions of linear regression (linearity, homoscedasticity, independence, and normality) were assessed and found to be acceptable. Multicollinearity was assessed by using the variance inflation factor (VIF) and also found to be acceptable. All analyses were conducted by using SAS Studio (SAS Institute Inc., Cary, NC, USA).

## 3. Results

*Subject selection:* There were 30,461 subjects available in the 2018 MEPS dataset. After application of the study eligibility criteria, a total of 2844 were included in the pain–hypercholesterolemia group, while 2724 were included in the pain–hypertension group. This resulted in a weighted 2018 population of 31,437,599 older US adults with pain and hypercholesterolemia, of which 8,592,982 were opioid users, and 29,187,826 older US adults with pain and hypertension, of which 8,143,470 were opioid users.

*Subject characteristics:* Table 1 outlines the predisposing, enabling, need, personal health practices, and external environmental factors of older US adults with pain and comorbid hypercholesterolemia or hypertension, respectively. In both groups, the majority of subjects were aged ≥65 years, female, and white. Most had completed higher than high school education, were unemployed, had private health insurance, middle/high income, <5 chronic conditions, no IADL limitation, no ADL limitation, no functional limitation, no work limitation, no visual limitation, no hearing limitation, little/moderate pain, good/very good/excellent health, and did not regularly exercise. The most common census region was the south. There were subgroup differences (*p* < 0.05) between opioid use and no opioid use for all characteristics except age in both groups (pain–hypercholesterolemia *p* = 0.2114; pain–hypertension *p* = 0.3604), education in the pain–hypertension group (*p* = 0.3941), health insurance in both groups (pain–hypercholesterolemia *p* = 0.0588; pain–hypertension *p* = 0.0766), visual limitation in the pain–hypercholesterolemia group (*p* = 0.1215), hearing limitation in both groups (pain–hypercholesterolemia *p* = 0.2927; pain–hypertension *p* = 0.2013), exercise in the pain–hypertension group (*p* = 0.5764), and region in both groups (pain–hypercholesterolemia *p* = 0.1012; pain–hypertension *p* = 0.1999).

*Descriptive healthcare expenditures:* Annual mean healthcare expenditures are reported in Table 2. Compared to non-users, expenditures were higher among opioid users for outpatient, office-based, prescription medication, other, and total healthcare expenditures in both groups. Inpatient expenditures were higher among opioid users than non-users in the pain–hypertension group (*p* = 0.0139) but not in the pain hypercholesterolemia group (*p* > 0.05). There was no difference between opioid users and non-users for emergency room expenditures in both groups (*p* > 0.05).

**Figure 1:**
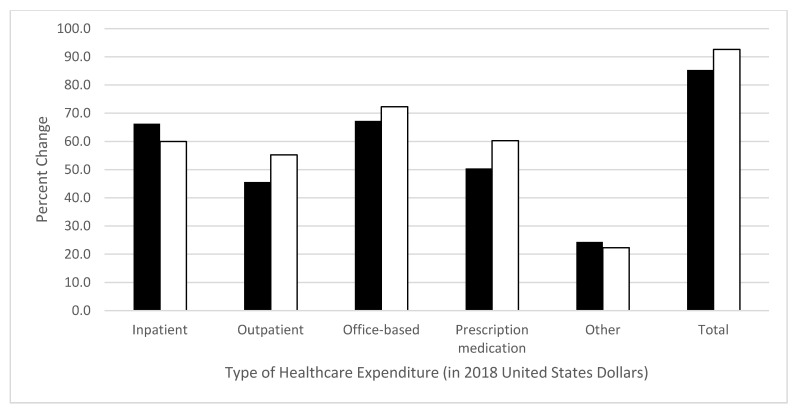
Percent difference in 2018 healthcare expenditures for opioid users versus non-users among United States adults age ≥50 years with pain in the past four weeks and a diagnosis of hypercholesterolemia or hypertension, after adjusting for predisposing, enabling, need, personal, and environmental factors. Percent change represents the difference in expenditure between opioid users and non-users, calculated by using semi-logarithmic equations (e^β^−1). Black bars represent the pain–hypercholesterolemia group. White bars represent the pain–hypertension group. The *p*-values for pain–hypercholesterolemia group: inpatient *p* = 0.0033, outpatient *p* < 0.0011, office-based *p* < 0.0001, prescription medication *p* < 0.0001, other *p* = 0.0056, total *p* < 0.0001, and emergency room *p* = 0.7068. The *p*-values for pain–hypertension group: inpatient *p* = 0.0043, outpatient *p* = 0.0006, office-based *p* < 0.0001, prescription medication *p* < 0.0001, other *p* = 0.0084, total *p* < 0.0001, and emergency room *p* = 0.1706.

*Adjusted healthcare expenditures:* The beta-estimates and standard errors from the adjusted linear regression models are shown in Table 3, and the percent difference in adjusted healthcare expenditures of opioid users versus non-users is shown in Figure 1. In the pain–hypercholesterolemia group, after adjusting for predisposing, enabling, need, personal health practices, and external environmental factors, opioid users had 66% greater inpatient expenditure (β = 0.51, *p* = 0.0033), 46% greater outpatient expenditure (β = 0.38, *p* < 0.0011), 67% greater office-based expenditure (β = 0.51, *p* < 0.0001), 50% greater prescription medication expenditure (β = 0.41, *p* < 0.0001), 24% greater other healthcare expenditure (β = 0.22, *p* = 0.0056), and 85% greater total healthcare expenditure (β = 0.62, *p* < 0.0001). There was no difference between opioid users and non-users for emergency room expenditures (β = −0.04, *p* = 0.7068).

Meanwhile, adjusted results in the pain–hypertension group found opioid users had 60% greater inpatient expenditure (β = 0.47, *p* = 0.0043), 55% greater outpatient expenditure (β = 0.44, *p* = 0.0006), 72% greater office-based expenditure (β = 0.54, *p* < 0.0001), 60% greater prescription medication expenditure (β = 0.47, *p* < 0.0001), 22% greater other healthcare expenditure (β = 0.20, *p* = 0.0084), and 93% greater total healthcare expenditure (β = 0.66, *p* < 0.0001). There was no difference between opioid users and non-users for emergency room expenditures (β = 0.16, *p* = 0.1706).

## 4. Discussion

The key finding from this study was that healthcare expenditures were greater among opioid users compared to non-users for all categories (inpatient, outpatient, office-based, prescription medication, other, and total) of healthcare expenditure, except emergency room costs (where there was no difference between groups) in this nationally representative sample of older US adults with pain and comorbid hypercholesterolemia or hypertension.

The findings of our study parallel those of previous studies; for example, a retrospective claims-based analysis of patients with long-term prescription opioid use showed significantly greater healthcare utilization and expenditures than matched non-users over one-year ($23,049 vs. $4975, *p* < 0.001) [26]. In our study, total annual healthcare expenditures were 85% and 93% greater among opioid users than non-users in both the pain–hypercholesterolemia and pain–hypertension groups respectively. The greater expenditures among opioid users may be explained by increased healthcare service utilization and disproportionate use of expensive healthcare services or increased risk of unintentional effects of opioid use, such as overdose [27]. It is also possible that the higher costs associated with opioid users could be attributed to the severity, type, or location of pain. Although pain severity was controlled for in the analysis, data on type or location of pain were unavailable in MEPS.

However, a strength of our study was the ability to assess several categories of healthcare expenditures. For example, our study found opioid users had 60–66% greater inpatient expenditures than non-users. This result compares favorably to the findings of a previous retrospective longitudinal cohort study of 20,201 commercially insured adults from 2006 to 2015 that showed that those using chronic opioid therapy were more likely to use inpatient services compared to non-users (adjusted odds ratio = 1.11; 95% CI = 1.01, 1.21) [28].

Another possible reason for greater healthcare expenditures among opioid users was the characteristics of the study subjects. There were significant differences in most characteristics between opioid users and non-users in both the pain–hypercholesterolemia and pain–hypertension groups. However, such statistical differences may be due to the large sample sizes.

Nevertheless, our study focused on opioid use among older adults, which poses pain-management challenges and may result in greater healthcare service use and costs. Older adults typically have a higher prevalence of pain [7,29], multiple chronic conditions [30,31], and disabilities [32], compared to younger adults, leading to higher use of opioids [33]. A retrospective study using data from the National Alzheimer’s Coordinating Center (2005–2017) among 13,059 older adult opioid users found that the most common comorbidity was hypertension (55.0%), which was a significant predictor of chronic opioid use [34]. The physiological changes associated with aging and an increased risk of falls, fractures [35], and delirium [36] makes older adults more vulnerable to adverse events of opioids that may result in more emergency room visits and hospital admissions. The risks of harm and subsequent expenditures may also be exacerbated by use of multiple healthcare providers and uncoordinated healthcare [37,38]. Further research could be conducted to identify the predictive characteristics of opioid use among older adults with pain and comorbid conditions, in order to target appropriate pain management interventions more effectively while simultaneously reducing costs and improving health outcomes.

*Clinical implications:* Although this study found healthcare expenditures of older adults with pain and comorbid conditions were greater among opioid users than non-users, we suggest this should not deter from the use of opioids when deemed clinically appropriate. For example, some older adults are unable to tolerate non-opioid analgesics such as non-steroidal anti-inflammatory drugs (NSAIDs), while the prolonged use of NSAIDs is not advised in older adults with hypertension, peptic ulcer disease, and impaired liver or renal function [39]. Furthermore, clinical guidelines and evidence from clinical trials have shown opioid therapy for older adults can be safe and effective when used with appropriate cautions, including lower starting doses, slower titration, longer dosing interval, and more frequent monitoring [40,41]. Higher rates of adverse effects are observed with initiation of opioids, but data also suggest that these may be preventable if monitored closely [42]. The use of opioid medications should therefore be considered as part of a holistic assessment of the patient’s pain management needs and comorbid conditions, and not healthcare expenditures alone.

*Study strengths and limitations:* Strengths of the study include the use of a nationally representative sample, offering improved generalizability of the results to the study population compared to previous studies; use of self-reported data to capture all information from the individual’s perspective; frequent survey administration and encouraged use of calendars and diaries to track healthcare use between interview surveys to reduce recall bias [43]; and provision of the most updated estimates of healthcare expenditures for opioid users versus non-users.

Limitations of the study include the broad definition of pain used (i.e., pain that interfered with normal work outside the home and housework in the past four weeks); an inability to calculate the true prevalence of opioid use, types of opioids used, dose and frequency of opioid, and reasons for opioid use, which might be important predictors of healthcare expenditures; inability to examine expenditures over the long-term due to the one-year study duration; and an inability to establish a cause and effect relationship due to the retrospective cross-sectional nature of the study design.

## 5. Conclusions

The findings of this study estimated that adjusted total healthcare expenditures were 85% to 93% greater among opioid users compared to non-users among older US adults with pain and comorbid hypercholesterolemia or hypertension. Future research is needed to identify the characteristics that can predict opioid use among these populations in order to help better understand opioid use and thus target appropriate pain management interventions more effectively.

## Figures and Tables

**Table 1:** Characteristics of United States older adults (age ≥ 50 years) with self-reported pain in the past four weeks and a diagnosis of hypercholesterolemia or hypertension.

		Pain–Hypercholesterolemia (N = 2844)			Pain–Hypertension (N = 2724)		
Characteristics		Opioid User (N = 787)	Non-User (N = 2057)	*p*	Opioid User (N = 762)	Non-User (N = 1962)	*p*
		Wt.% (95% CI)	Wt. % (95% CI)		Wt.% (95% CI)	Wt.% (95% CI)	
**Predisposing Factors:**							
Age (years)				0.2114			0.3604
	50–64	43.7 (39.7–47.7)	40.7 (37.7–43.6)		42.0 (37.8–46.1)	39.7 (36.8–42.5)	
	≥65	56.3 (52.3–60.3)	59.3 (56.4–62.3)		58.0 (53.9–62.2)	60.4 (57.5–63.2)	
Gender				0.0175			0.0234
	Male	42.8 (39.7–45.9)	47.4 (44.7–50.2)		41.3 (37.3–45.3)	46.7 (44.3–49.0)	
	Female	57.2 (54.1–60.3)	52.6 (49.9–55.3)		58.7 (54.7–62.7)	53.3 (51.0–55.7)	
Race				0.0062			0.0021
	White	85.3 (82.6–88.0)	80.6 (78.2–83.0)		83.1 (79.9–86.1)	77.4 (75.0–79.9)	
	Other	14.7 (12.0–17.4)	19.4 (17.0–21.8)		16.9 (13.8–20.1)	22.6 (20.0–25.0)	
**Enabling Factors:**							
Education				0.0130			0.3941
	High school or less	50.3 (46.1–54.5)	49.7 (45.5–53.9)		49.9 (45.4–54.5)	50.1 (45.5–54.6)	
	Higher than high school	44.6 (41.9–47.3)	55.4 (52.7–58.1)		47.8 (44.9–50.7)	52.2 (49.3–55.1)	
Employment				0.0004			0.0007
	Employed	26.9 (22.7–31.2)	35.0 (31.9–38.0)		25.6 (21.6–29.6)	33.5 (30.3–36.8)	
	Unemployed	73.1 (68.8–77.3)	65.0 (62.0–68.1)		74.4 (70.4–78.4)	66.5 (63.2–69.7)	
Health insurance				0.0588			0.0766
	Private	52.4 (48.3–56.5)	56.6 (53.1–60.2)		51.8 (47.3–56.3)	53.9 (50.8–57.1)	
	Public	46.8 (42.8–50.9)	41.8 (38.2–45.4)		47.7 (40.0–48.6)	44.4 (41.2–47.6)	
	Uninsured	0.8 (0.2–1.4)	1.6 (1.0–2.2)		0.5 (0.0–1.0)	1.6 (1.0–2.2)	
Poverty status				0.0005			0.0148
	Poor/near poor/low income	38.8 (34.2–43.5)	30.3 (27.7–32.9)		39.2 (34.7–43.8)	33.2 (30.4–36.0)	
	Middle/high income	61.2 (56.5–65.8)	69.7 (67.1–72.3)		60.8 (56.2–65.3)	66.8 (64.0–69.6)	
**Need Factors:**							
Chronic conditions				<0.0001			<0.0001
	<5	69.3 (55.5–73.1)	80.8 (78.7–83.0)		68.5 (64.2–72.8)	79.5 (77.5–81.6)	
	≥5	30.7 (26.9–34.5)	19.2 (17.0–21.3)		31.5 (27.2–35.8)	20.5 (18.4–22.5)	
IADL limitation				<0.0001			<0.0001
	Yes	13.8 (11.1–16.5)	8.8 (7.5–10.1)		16.2 13.3–19.1)	10.6 (9.0–12.1)	
	No	86.2 (83.5–88.9)	91.2 (89.9–92.5)		83.8 (80.9–86.7)	89.4 (87.9–91.0)	
ADL limitation				0.0100			0.0043
	Yes	7.5 (5.6–9.3)	4.9 (3.7–6.0)		9.0 (6.5–11.5)	5.8 (4.6–7.1)	
	No	92.5 (90.7–94.4)	95.1 (94.0–96.3)		91.0 (88.5–93.5)	94.2 (92.9–95.4)	
Functional limitation				<0.0001			<0.0001
	Yes	58.4 (54.2–62.5)	38.9 (36.2–41.6)		63.5 (59.1–67.9)	41.6 (38.9–44.3)	
	No	41.6 (37.5–45.8)	61.1 (58.4–63.8)		36.5 (32.1–40.9)	58.4 (55.7–61.1)	
Work limitation				<0.0001			<0.0001
	Yes	46.1 (42.3–50.0)	25.9 (23.6–28.2)		49.4 (44.8–54.0)	28.2 (25.7–30.6)	
	No	53.9 (50.0–57.7)	74.1 (71.8–76.4)		50.6 (46.0–55.2)	71.8 (69.4–74.3)	
Visual limitation				0.1215			0.0066
	Yes	8.7 (6.3–11.1)	6.6 (5.3–8.0)		10.3 (7.7–12.9)	6.7 (5.3–8.0)	
	No	91.3 (88.9–93.7)	93.4 (92.0–94.7)		89.7 (87.1–92.3)	93.3 (92.0–94.7)	
Hearing limitation				0.2927			0.2013
	Yes	17.0 (13.9–20.2)	15.3 (13.5–17.1)		17.5 (14.2–20.8)	15.3 (13.5–17.1)	
	No	83.0 (79.8–86.1)	84.7 (82.9–86.5)		82.5 (79.2–85.8)	84.7 (82.9–86.5)	
Pain				<0.0001			<0.0001
	Little	30.5 (26.4–34.5)	55.3 (52.6–58.0)		27.6 (23.7–31.6)	51.5 (48.9–54.2)	
	Moderate	26.1 (22.4–29.9)	21.9 (19.8–23.9)		24.7 (21.2–28.2)	23.0 (20.7–25.3)	
	Quite a bit	29.9 (25.9–34.0)	17.9 (16.0–19.8)		33.5 (29.4–37.7)	19.3 (17.4–21.3)	
	Extreme	13.5 (10.7–16.2)	4.9 (3.9–5.9)		14.2 (11.4–16.9)	6.1 (5.0–7.3)	
Perceived health status				<0.0001			<0.0001
	Excellent/very good/good	60.6 (56.2–65.1)	73.0 (70.4–75.7)		57.9 (53.4–62.4)	71.2 (68.6–73.7)	
	Fair/poor	39.4 (34.9–43.8)	27.0 (24.3–29.6)		42.1 (37.6–46.6)	28.8 (26.3–31.4)	
**Personal Health Practices Factors:**							
Exercise				0.0050			0.5764
	Yes	34.6 (30.6–38.6)	40.8 (37.7–44.0)		36.4 (31.9–41.0)	37.9 (34.8–41.0)	
	No	65.4 (61.4–69.4)	59.2 (56.0–62.3)		63.6 (59.0–68.1)	62.1 (59.0–65.2)	
**External Environmental Factors:**							
Census region				0.1012			0.1999
	Northeast	14.7 (10.3–19.1)	18.9 (16.4–21.4)		16.1 (11.5–20.7)	18.7 (16.2–21.2)	
	Midwest	21.3 (17.6–24.9)	20.4 (17.9–23.0)		22.7 (18.8–26.6)	21.5 (18.8–24.2)	
	South	44.8 (39.9–49.8)	39.4 (36.8–42.0)		44.0 (39.1–48.9)	39.4 (36.5–42.3)	
	West	19.2 (16.2–22.3)	21.3 (18.9–23.6)		17.2 (13.8–20.6)	20.4 (17.9–22.9)	

Wt.% = weighted percent. CI = confidence interval. IADL = instrumental activities of daily living. ADL = activities of daily living. Study subjects consisted of United States adults alive during the calendar year 2018, age ≥50 years, with self-reported pain in the past four weeks, and a diagnosis of hypercholesterolemia (pain–hypercholesterolemia group) or hypertension (pain–hypertension group). The *p*-value indicates differences between opioid users and non-users for each variable in both the pain–hypercholesterolemia and pain–hypertension groups, based on chi-square tests.

**Table 2:** Descriptive healthcare expenditures of United States older adults (age ≥50 years) with self-reported pain in the past four weeks and a diagnosis of hypercholesterolemia or hypertension.

	Pain–Hypercholesterolemia (N = 2844)			Pain–Hypertension (N = 2724)		
Healthcare Expenditures	Opioid User	Non-User	*p*	Opioid User	Non-User	*p*
	Mean (standard error)	Mean (standard error)		Mean (standard error)	Mean (standard error)	
Inpatient	24,676 (3080)	18,045 (1415)	0.0569	25,185 (2903)	16,877 (1268)	0.0139
Outpatient	5085 (716)	2602 (229)	0.0015	5142 (691)	2724 (197)	0.0007
Office-based	5666 (383)	3158 (160)	<0.0001	5456 (386)	3094 (137)	<0.0001
Emergency room	1456 (150)	1510(132)	0.7829	1611 (154)	1369 (109)	0.2146
Prescription medications	5498 (349)	3736 (210)	<0.0001	6024 (371)	3998 (245)	<0.0001
Other	3637 (308)	2626 (200)	0.0062	4017 (403)	2880 (196)	0.0104
Total	25,398 (1488)	12,556 (448)	<0.0001	26,457 (1448)	13,100 (456)	<0.0001

Study subjects consisted of United States adults alive during the calendar year 2018, age ≥50 years, with self-reported pain in the past four weeks, and a diagnosis of hypercholesterolemia (pain–hypercholesterolemia group) or hypertension (pain–hypertension group), but only included those who had positive healthcare expenditures for each healthcare expenditure category. The *p*-value indicates differences between opioid users and non-users for each healthcare expenditure category in both the pain–hypercholesterolemia and pain–hypertension groups, based on *t*-tests.

**Table 3:** Adjusted parameter estimates using logged positive healthcare expenditures of United States older adults (age ≥50 years) with self-reported pain in the past four weeks and a diagnosis of hypercholesterolemia or hypertension.

		Pain–Hypercholesterolemia (N = 2844)		Pain–Hypertension (N = 2724)	
Healthcare Expenditures		Beta Estimate (Standard Error)	*P*	Beta Estimate (Standard Error)	*p*
Inpatient					
	Intercept	6.68 (0.97)	<0.0001	7.47 (0.68)	<0.0001
	Opioid user	0.51 (0.17)	0.0033	0.47 (0.16)	0.0043
	Non-user	Reference		Reference	
Outpatient					
	Intercept	7.07 (0.64)	<0.0001	6.48 (0.74)	<0.0001
	Opioid user	0.38 (0.11)	0.0011	0.44 (0.13)	0.0006
	Non-user	Reference		Reference	
Office-based					
	Intercept	7.04 (0.34)	<0.0001	6.73 (0.30)	<0.0001
	Opioid user	0.51 (0.06)	<0.0001	0.54 (0.06)	<0.0001
	Non-user	Reference		Reference	
Emergency room					
	Intercept	6.51 (0.63)	<0.0001	6.19 (0.73)	<0.0001
	Opioid user	−0.04 (0.11)	0.7068	0.16 (0.12)	0.1706
	Non-user	Reference		Reference	
Prescription medications					
	Intercept	6.80 (0.37)	<0.0001	6.94 (0.35)	<0.0001
	Opioid user	0.41 (0.07)	<0.0001	0.47 (0.08)	<0.0001
	Non-user	Reference		Reference	
Other					
	Intercept	6.16 (0.36)	<0.0001	6.34 (0.37)	<0.0001
	Opioid user	0.22 (0.08)	0.0056	0.20 (0.08)	0.0084
	Non-user	Reference		Reference	
Total					
	Intercept	8.49 (0.29)	<0.0001	8.20 (0.33)	<0.0001
	Opioid user	0.62 (0.05)	<0.0001	0.66 (0.05)	<0.0001
	Non-user	Reference		Reference	

Study subjects consisted of United States adults alive during the calendar year 2018, age ≥ 50 years, with self-reported pain in the past four weeks, and a diagnosis of hypercholesterolemia (pain–hypercholesterolemia group) or hypertension (pain–hypertension group). Models adjusted for the following variables: age, gender, race, education, employment, health insurance, poverty status, chronic conditions, instrumental activities of daily living limitation, activities of daily living limitation, functional limitation, work limitation, visual limitation, hearing limitation, pain, perceived health status, exercise, and census region. Pain–hypercholesterolemia group R^2^ values: inpatient = 0.15, outpatient = 0.05, office-based = 0.12, emergency room = 0.07, prescription medication = 0.19, other = 0.17, and total = 0.25. Pain–hypertension group R^2^ values: inpatient = 0.14, outpatient = 0.07, office-based = 0.13, emergency room = 0.07, prescription medication = 0.17, other = 0.20, and total = 0.25.

## Data Availability

The data presented in this study are available on request from the corresponding author.
